# Drug Repurposing, an Attractive Strategy in Pancreatic Cancer Treatment: Preclinical and Clinical Updates

**DOI:** 10.3390/cancers13163946

**Published:** 2021-08-05

**Authors:** Laura De Lellis, Serena Veschi, Nicola Tinari, Zhirajr Mokini, Simone Carradori, Davide Brocco, Rosalba Florio, Antonino Grassadonia, Alessandro Cama

**Affiliations:** 1 Department of Pharmacy, G. d’Annunzio University of Chieti-Pescara, 66100 Chieti, Italy; serena.veschi@unich.it (S.V.); simone.carradori@unich.it (S.C.); davide.brocco@unich.it (D.B.); rosalba.florio@unich.it (R.F.); 2 Department of Medical, Oral and Biotechnological Sciences, G. d’Annunzio University of Chieti-Pescara, 66100 Chieti, Italy; ntinari@unich.it (N.T.); grassadonia@unich.it (A.G.); 3 Center for Advanced Studies and Technology—CAST, G. d’Annunzio University of Chieti-Pescara, 66100 Chieti, Italy; 4 European Society of Anaesthesiology and Intensive Care (ESAIC) Mentorship Programme, ESAIC, 24 Rue des Comédiens, BE-1000 Brussels, Belgium; zhirajrmokini@yahoo.com

**Keywords:** pancreatic ductal adenocarcinoma, drug repositioning, repurposed drugs, tumor microenvironment, immunomodulation

## Abstract

**Simple Summary:**

The purpose of this review is to provide an update on some of the most promising non-oncology drugs that are already approved and that could be useful also in the treatment of pancreatic cancer, one of the deadliest malignancies worldwide. Current chemotherapy options are unsatisfactory in this cancer and there is an urgent need for more effective and less toxic drugs to improve the dismal pancreatic cancer therapy. The use in cancer therapy of drugs approved for other indications (drug repurposing) is an attractive approach that has the potential to overcome several issues associated with de novo drug discovery, such as dose-finding and safety profiles, accelerating their clinical adoption. In this review, we report proposed mechanisms of action and biological targets of drugs that are candidates for repurposing in pancreatic cancer therapy, focusing on targets that appear to be relevant for their anticancer action. Finally, considering that cancer immunotherapy provides remarkable long-term remission in some responsive tumors and that this strategy is mostly ineffective in pancreatic cancer patients, we discuss recent developments regarding the ability of some repurposing drug candidates to activate anti-tumor immune response, which may be particularly relevant for their clinical effectiveness.

**Abstract:**

Pancreatic cancer (PC) is one of the deadliest malignancies worldwide, since patients rarely display symptoms until an advanced and unresectable stage of the disease. Current chemotherapy options are unsatisfactory and there is an urgent need for more effective and less toxic drugs to improve the dismal PC therapy. Repurposing of non-oncology drugs in PC treatment represents a very promising therapeutic option and different compounds are currently being considered as candidates for repurposing in the treatment of this tumor. In this review, we provide an update on some of the most promising FDA-approved, non-oncology, repurposed drug candidates that show prominent clinical and preclinical data in pancreatic cancer. We also focus on proposed mechanisms of action and known molecular targets that they modulate in PC. Furthermore, we provide an explorative bioinformatic analysis, which suggests that some of the PC repurposed drug candidates have additional, unexplored, oncology-relevant targets. Finally, we discuss recent developments regarding the immunomodulatory role displayed by some of these drugs, which may expand their potential application in synergy with approved anticancer immunomodulatory agents that are mostly ineffective as single agents in PC.

## 1. Introduction

Pancreatic cancer (PC) is one of the deadliest malignancies worldwide and it is predicted to become the second leading cause of cancer-related death in Western countries within the next 10 years [1]. PC is characterized by a 5-year survival rate of only 9% and this poor prognosis is mainly due to a late diagnosis, since patients rarely display symptoms until an advanced and unresectable stage of the disease. Moreover, improvements in PC therapeutic options and survival have been limited over the past decades, as compared to other tumors; thus, an unsatisfactory response to standard chemotherapy still remains an unresolved problem [2]. At present, gemcitabine, together with combined treatments including FOLFIRINOX and gemcitabine/nab-paclitaxel, are the mainstay treatment regimens for PC, but they are rather toxic and globally offer a limited advantage in overall survival [3]. In addition, potential benefits of new agents targeting specific aberrations, such as *BRCA1/BRCA2* mutations or *NTRK1-3* fusion genes, are limited to small subgroups of patients [4]. Similarly, treatments based on immune checkpoint inhibitors (ICIs) may be potentially beneficial in PC patients with defective mismatch repair (MMR), who ideally have highly immunogenic tumors, but unfortunately, only modest results were obtained in such patients [5]. Radical surgery is the only potentially curative choice for PC, but only a small fraction of patients (15–20%) are diagnosed at a resectable stage of the disease and most patients relapse after surgery [6]. Hence, novel, more effective and less toxic drugs are needed to improve the dismal PC therapy.

The development of new anti-cancer drugs is an expensive and time-consuming process that requires extensive and robust cell- and animal-based studies, followed by safety and efficacy testing in humans by means clinical trials to substantiate preclinical results [7]. It is worth noting that this process, which takes an average time of 13 years, is very challenging because a promising new chemical entity may fail in proceeding through the clinical trial phases needed before its approval as a drug, due to the emergence of unpredicted safety problems, or the lack of effectiveness in patients [7,8,9]. In such a scenario, repurposing of approved drugs in cancer therapy is an attractive and alternative approach that has the potential to overcome several issues associated with de novo drug discovery [10,11,12]. In this regard, one of the main strengths of this approach is that candidates for drug repurposing have a well-documented history of clinical use for the original indication, thus, pharmacokinetic, pharmacodynamic, dosing and toxicity profiles have been already established. This may accelerate their clinical development, because in principle, it may directly start from phase II clinical trials to assess the efficacy for the new indication/disease target, thereby reducing the risk of failure for candidate agents. Moreover, fewer investments appear to be required for the implementation of a repurposed drug in clinical practice [13], although phase III and regulatory costs are likely to remain quite similar to those for a novel drug in the same indication [11].

The first successful examples of drug repurposing largely resulted from serendipitous discoveries, whereas subsequent systematic strategies emerged for the identification of non-oncology drugs to be potentially repurposed in cancer therapy [10,11]. They may be essentially grouped into two categories: computational and experimental approaches [10,11]. The first rely on high-throughput analyses combined with bioinformatic tools (e.g., pathway/network mapping, signature matching, molecular docking), or the systematic evaluation of electronic health records (EHRs), as in the case of retrospective clinical analyses. The second are mainly activity-based and employ binding assays (e.g., proteomic techniques, chemical genetic approaches) to identify relevant interactions among novel targets and known drugs, or cell-based phenotypic screening centered on the selection of common phenotypic criteria (e.g., proliferation, modulation of exosome biogenesis, cell cycle profiling), without a priori knowledge of the target affected [11,14]. At present, there are several non-oncology drugs that have been successfully repurposed in cancer therapy [11]. For instance, thalidomide originally prescribed as a treatment for morning sickness in pregnant women was withdrawn from the market because of severe teratogenic side effects, but it was serendipitously found to be effective first in the treatment of erythema nodosum leprosum (ENL); next, it was reauthorized in 2006 as first-line approach in elderly patients with advanced multiple myeloma in combination with melphalan and prednisone [15]. Similarly, the beta-blocker (BB) propranolol used for cardiovascular disease has been widely used off-label to treat infantile hemangiomas (IHs) since its effectiveness was serendipitously reported in 2008, before receiving FDA approval in 2014 as a unique oral pediatric formulation for IH therapy [16,17]. Another example is raloxifene that belongs to the class of selective estrogen receptor modulators (SERMs) and was originally approved for the prevention and treatment of osteoporosis in postmenopausal women. In 2007, this drug was effectively repurposed after retrospective analyses of clinical trial data and received FDA approval for the treatment of post-menopausal women with ER+/PR+ advanced breast cancer [18]. Based on these considerations, development, marketing and clinical adoption of low-cost, already-approved non-oncology drugs to be employed in cancer treatment represent a valuable opportunity, especially in cases of hard-to-treat, relatively rare malignancies, including pancreatic cancer [11,19].

**Figure 1:**
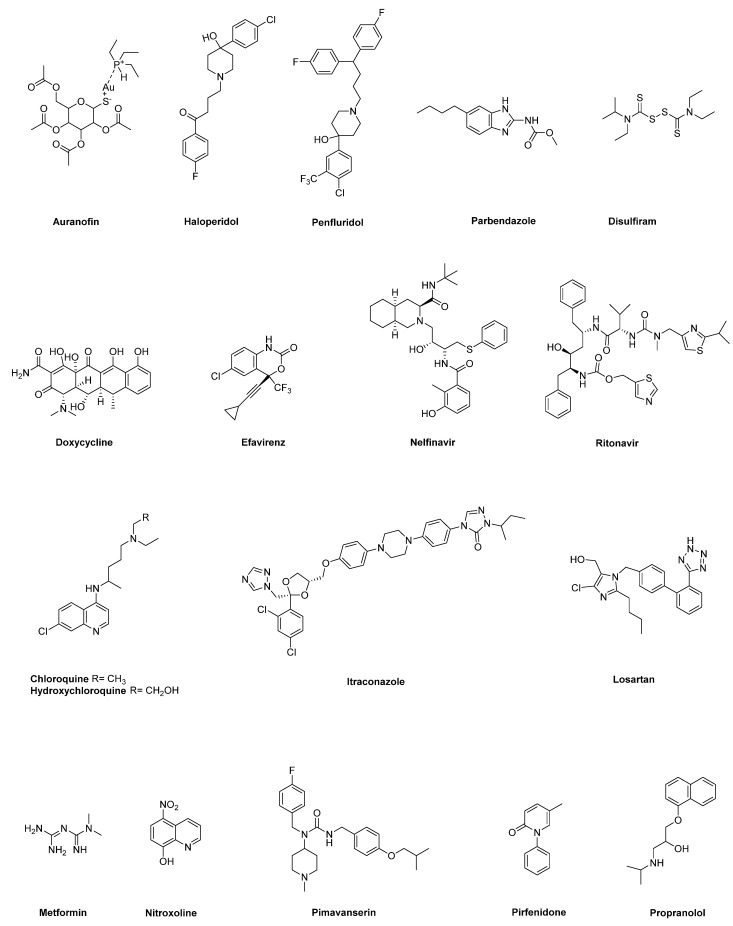
Chemical structure of repurposed drug candidates in pancreatic cancer treatment discussed in the review.

In this review, we provide an update on some of the most promising repurposed drug candidates (Figure 1) that show prominent clinical and preclinical data in pancreatic cancer, with a focus on molecular mechanisms and targets modulated by these non-oncology drugs to expand our insights into their therapeutic relevance. For some drugs lacking in vivo, or clinical studies in PC, we outline the most significant data shown by these drugs in other tumors, in view of their potential value as repurposing candidates in the treatment of PC.

## 2. Repurposing Drug Candidates in Pancreatic Cancer: Preclinical and Clinical Findings

**Figure 2:**
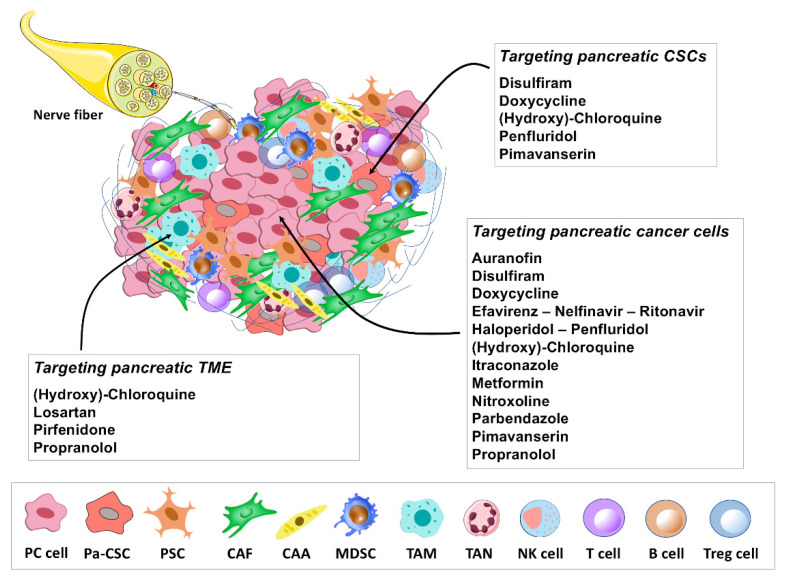
Overview of the preferential targeting shown by repurposing drug candidates in pancreatic cancer. The figure depicts the complex PC tumor microenvironment characterized by a tight crosstalk among PC cells and cell components within the PC-associated stroma. Repurposing drug candidates discussed in the review are grouped on the basis of preclinical evidence indicating their ability to modulate tumor microenvironment (TME) components, pancreatic cancer (PC) cells and/or pancreatic cancer stem cells (CSCs). Proposed molecular targets/mechanisms of antitumor action in PC for repurposing drug candidates are detailed in Table 1. Pancreatic cancer stem cell, Pa-CSC; pancreatic stellate cell, PSC; cancer-associated fibroblast; cancer-associated adipocyte, CAA; myeloid-derived suppressor cell, MDSC; tumor-associated macrophage, TAM; tumor-associated neutrophil, TAN; natural killer, NK; regulatory T cells, Treg.

A growing number of studies reported encouraging data about antitumor effects displayed by FDA-approved non-oncology drugs in PC. In this section, we review updated preclinical and clinical studies supporting the relevance of different non-oncology drugs, belonging to disparate therapeutic categories, as candidates for repurposing in PC therapy (Table 1 and Table 2 and Figure 2). For literature review, the Medline database (https://pubmed.ncbi.nlm.nih.gov/, accessed on April 2021) was searched by combining several keywords, such as “pancreatic cancer”, “repurposed drug”, “drug repurposing” and “drug repositioning”. In addition, the information about clinical data for each candidate was searched via the website “https://clinicaltrials.gov/”, accessed on May 2021. For drugs lacking studies in PC animal models, the NCI Developmental Therapeutics Program (NCI/DTP) database was also accessed (https://dtp.cancer.gov/, accessed on July 2021), to search for in vivo results in other cancer models. To provide a balanced review of some of the most promising non-oncology drugs candidates for repurposing in PC therapy, both preclinical and clinical studies included and discussed in the review have been selected by the authors for their relevance to the topic.

### 2.1. Auranofin

Auranofin is an orally available organogold complex presenting an Au-S bond stabilized by a triethylphosphine group (Figure 1). This compound has been approved for the treatment of rheumatoid arthritis because it inhibits inflammation, lysosomal enzyme release and phagocytosis by macrophages, thereby slowing down progression of the disease [92]. Interestingly, several studies described anticancer effects of auranofin in different tumors, including pancreatic cancer, as single drug, or in combination [20,21,22,93,94,95]. In particular, a first study showed that auranofin affects the viability of multiple PC cell lines with IC_50_ values all below 5 μmol/L, possibly through the inhibition of one of the major redox systems, namely thioredoxin reductase 1 (TrxR1), which in turn leads to decreased antioxidant activity within cancer cells, followed by apoptosis induction [20]. The authors showed that auranofin may also inhibit the hypoxia-inducible factor-1 α (HIF1α) as an additional mechanism of antitumor action [20]. Ex vivo and in vivo studies substantiated in vitro findings, showing antitumor effect of auranofin at the primary site and complete suppression of metastasis at distant organs [20]. In line with these results, Onodera et al. [21] reported that auranofin displays preferential cytotoxicity towards PC cells under nutrient-deprived as compared with nutrient-sufficient conditions, through mechanisms involving ROS accumulation and subsequent induction of apoptosis by caspase 3/7 activation and proteolytic PARP cleavage [21]. Auranofin was also able to suppress tumor growth in a PC xenograft model, with a potency comparable with that of cisplatin and no adverse effects in the animals [21]. Interestingly, in several PC cells with distinct genetic backgrounds auranofin promoted the increase in mitochondrial ROS and apoptosis, as well as the inhibition of autophagic flux when used in combination with an engineered human cyst(e)inase, which was previously shown to lead to oxidative stress and selective cancer cell cytotoxicity in multiple murine tumor models by depletion of extracellular pools of L-Cys [22,96]. Moreover, the combined treatment of auranofin with cyst(e)inase synergistically suppressed the growth of PC xenografts, without systemic toxicity, providing a rationale to explore therapeutic strategies targeting multiple antioxidant pathways in the perspective of clinical translation [22].

Despite the promising preclinical results shown by auranofin, it should be noted that at present, this drug is rarely used for the original indication due to the emergence of novel antirheumatic medications; thus, it has been discontinued from several markets in the world and this may hamper its supply for clinical trials, as well as its potential clinical adoption [19].

No clinical trial of auranofin in PC has been performed. However, the drug has been tested in a small pilot study in asymptomatic ovarian cancer patients with marker elevation and found to be safe [97]. Moreover, a phase I–II trial in Chronic Lymphocytic Leukemia was undertaken, but results were not provided (NCT01419691).

### 2.2. Anti-Psychotic Drugs (Haloperidol, Penfluridol)

Haloperidol and penfluridol are hydroxypiperidine-based anti-psychotic drugs (Figure 1) that are employed to control symptoms associated with several psychotic disorders, mainly through the blockade of dopamine D2 receptor (DRD2) [98]. Interestingly, these drugs have been reported to have relevant antitumor properties in several in vitro and in vivo tumor models [99]. Using pathway- and network-based approaches combined with PC transcriptome profiles and by comparing healthy pancreatic tissue samples to a large set of PC tumors, Jandaghi et al. showed that DRD2 expression is increased in PDAC, where it plays a key role in PC cell proliferation and survival [23]. Accordingly, the pharmacologic blockade of DRD2 activity by haloperidol suppressed PC cell proliferation by promoting endoplasmic reticulum (ER) stress, with minimal toxicity on normal fibroblasts. Haloperidol also affected migration, hampered cell cycle progression and induced apoptosis of PC cells. Remarkably, when administrated to mice with orthotopic xenograft PC tumors, haloperidol significantly reduced tumor size and metastatic dissemination, while showing no effects on animal weights [23]. A distinct mechanism through which haloperidol may exert its antitumor activity in PC was proposed by Kim and co-workers [24]. Haloperidol showed to affect MIA PaCa-2 pancreatic cancer cell viability at least in part by restoring the expression of dual-specificity phosphatase 6 (DUSP6) gene, which is a phosphatase selective for extracellular signal-regulated kinase 1/2 (ERK1/2). In fact, DUSP6 is implicated as a tumor suppressor gene in PC [100] and, specifically, its expression is suppressed in MIA PaCa-2 cells through intron 1 hypermethylation. In such a scenario, haloperidol was shown to be able to promote DNA demethylation of the DUSP6 gene in MIA PaCa-2 cells. Considering that DUSP6 affects ERK signaling through dephosphorylation, or nuclear translocation inhibition [101], and that ERK signaling blockade suppresses proliferation and invasion of cancer cells, the epigenetic modification in transcriptional regulation of DUSP6 gene promoted by haloperidol in PC cells appears to contribute to its antiproliferative activity. As far as penfluridol, it showed antiproliferative effects in distinct PC cell lines through mechanisms related to the induction of endoplasmic reticulum (ER) stress, leading to autophagy and subsequent PC cell apoptosis [25,26]. In vitro findings were confirmed both in xenograft and orthotopic PC models, where penfluridol significantly reduced pancreatic tumor growth [25,26]. As an additional antiproliferative mechanism, penfluridol was reported to affect PC growth by promoting cell cycle arrest and apoptosis via activation of protein phosphatase 2 (PP2A) [27], whose stimulation has been reported to inhibit pancreatic cancer tumorigenicity both in vitro and in vivo [102]. Interestingly, penfluridol was also able to synergize with gemcitabine in affecting viability of both gemcitabine-resistant and gemcitabine-sensitive PC cells [27]. More recently, Dandawate and co-workers showed that prolactin receptor (PRLR)-based signaling is active in PC and proposed a novel mechanism through which penfluridol may affect PC growth [28]. In fact, penfluridol was shown to target the JAK2 binding site in PRLR, thereby suppressing JAK2–STAT3 and ERK/AKT signaling, which are key players in cancer cell proliferation, migration, apoptosis and in conferring stem-cell features. As a consequence, penfluridol suppressed colony and spheroid formation, together with the induction of autophagy, thereby inhibiting PC cell proliferation. Moreover, penfluridol was able to slow down the growth of different xenograft mouse models of PC [28], supporting its potential repurposing in PC treatment.

As far as clinical trials, no studies have been reported in PC, and in general, there is a lack of clinical studies regarding anti-psychotic drugs in cancer, with the exception of dihydrolenperone in lung cancer [103].

### 2.3. Benzimidazole-Based Anthelminthics

Benzimidazole-based anthelmintics are a family of drugs widely employed both in human and in veterinary medicine for the treatment of intestinal parasites [104]. Interestingly, there is a growing number of studies reporting that several members of this family display both in vitro and in vivo antitumor effects in multiple tumor models, including PC [29,105,106,107]. In particular, Florio et al. showed that parbendazole (Figure 1), at dosages in the range of the therapeutic plasma concentrations, inhibits growth and abolishes clonogenicity of PC cells by fostering apoptosis, drastic cell cycle perturbation and DNA damage response [29]. Mechanistically, parbendazole is able to profoundly alter the organization of microtubules in PC cells, which in turn promotes the formation of irregular mitotic spindles and the rapid appearance of polyploid cells. Notably, the combined treatment of parbendazole with the PC standard chemotherapeutic drug gemcitabine synergistically affects PC cell viability, supporting the relevance of parbendazole in the perspective of clinical translation. Intriguingly, considering that pharmaceutically active compounds may modulate multiple molecular targets with a plethora of mechanisms of action, putative polypharmacological profiles of a wider series of benzimidazoles were explored by using an in silico target prediction approach [106]. Notably, for the two derivatives fenbendazole and mebendazole, the bioinformatic tool highlighted a few previously underexplored cancer-related targets having very high probability scores, namely MAP kinase p38 alpha, vascular endothelial growth factor receptor 2 (VEGFR2) and the tyrosine-protein kinase ABL [106]. Further in vitro and in vivo studies in PC and also in additional tumor models are needed to validate these predictions, in view of the potential repurposing of benzimidazole-based anthelmintics in oncology.

At least two early phase trials tested albendazole in advanced cancer, mostly colorectal [108,109], and found that it was well tolerated. In one of these, two patients with advanced pancreatic cancer were enrolled [108].

### 2.4. Disulfiram

Disulfiram (DSF), an organic disulfide deriving from the oxidative dimerization of N,N-diethyldithiocarbamic acid (Figure 1), has been widely used in the clinic for the treatment of chronic alcoholism. Mechanistically, this drug irreversibly inhibits the aldehyde dehydrogenase (ALDH) enzyme, resulting in the accumulation of acetaldehyde, which in turn triggers unpleasant symptoms when alcohol is consumed [110]. Interestingly, several studies reported a promising antitumor role of DSF as a single agent, or in combined treatments, through the interference with ALDH-related processes of cell metabolism, which are strictly related with a stemness behavior of cancer cells [111]. Moreover, DSF may chelate copper divalent cations (Cu^2+^) to form DSF/Cu complexes that act as potent proteasome inhibitors, which in turn prevent the activation of NF-kB, a key transcription factor involved in the maintenance of cancer stem cell (CSC) biology [112]. Notably, these pathways proved to be modulated by DSF also in PC. Han et al. showed that diethyldithiocarbamate (DDTC), the major metabolite of DSF, in the presence of copper forms a binuclear complex DDTC-Cu(I) that affects PC cell proliferation in vitro and tumor growth in vivo by inhibiting proteasome activity. In this regard, treatment of mice harboring SW1990 PC cell xenografts with DDTC-Cu(I) showed accumulation of ubiquitinated proteins, together with the up-regulation of p27 and the down-regulation of NF-kB expression in tumor tissues [30]. As far as the targeting of PC stem cells, DSF/Cu showed to be effective in depleting pre-existing CSCs and radiation-induced CSCs in a panel of PC cell lines via NF-kB-stemness gene pathway downregulation. Furthermore, the combination of DSF, 5-FU and radiotherapy was the most effective in controlling growth in a syngeneic mouse PC model, as compared with chemo-radiotherapy alone, with no toxicity for the animals, and this pronounced antitumor effect was associated with reduced sphere formation, thereby supporting the potential of DSF in blocking therapy-induced stemness [31]. Another study showed that DSF may promote the switch from apoptosis to aponecrosis death pathways as an alternative form of cell death in PC cell lines with mutated Ras when employed in combination with clinically achievable concentrations of arsenic trioxide and ascorbic acid [32]. These effects, which are associated with intracellular ATP depletion and release of reactive oxygen species (ROS), were supported also by in vivo experiments, where the three-drug combination markedly affected tumor growth in mice with PANC-1 xenografts [32]. Interestingly, Zhang et al. explored an additional mechanism of action for further explaining the antitumor potential of DSF in PC [33]. The authors showed that DSF/Cu may foster cytotoxic autophagy-dependent apoptosis in PC cells through the activation of the ER stress/IRE1α-XBP1 pathway [33]. This activation may occur either by a direct interaction with IRE1α, or by an indirect mechanism involving the inhibition of the NPL4 cofactor of the p97/VCP segregase and of proteasome, along with the production of ROS [33].

Recently, the results of a phase I study of fixed-dose disulfiram (250 mg orally per day) and dose-escalating copper in patients with heavily pre-treated, metastatic cancer to the liver have been published. Among the 16 evaluable patients, no dose-limiting toxicity was observed, along with four patients exhibiting stable disease. The only patient with PC experienced disease progression [113]. Currently, two clinical trials are testing disulfiram in patients with PC. The first one is a partially randomized phase I study exploring disulfiram in association to chemotherapy. Eligible patients will have refractory cancers receiving chemotherapy at the discretion of the treating oncologist, or metastatic PC treated with gemcitabine. With an estimated accrual of 74 patients, the study aims at establishing maximum tolerated dose of disulfiram, and verifying whether the drug is able to limit muscle waste and increase cancer sensitivity to chemotherapy (NCT02671890). Disulfiram is also being tested with copper gluconate in a phase II pilot trial in patients with metastatic PC receiving FOLFIRINOX, Gemcitabine+nab-Paclitaxel, or single agent Gemcitabine, with rising levels of CA19.9, but no radiologic sign of disease progression. Primary endpoint of the study is the assessment of changes in plasma levels of CA19.9, while secondary endpoints are efficacy related (NCT03714555).

### 2.5. Doxycycline

Doxycycline is a semi-synthetic broad-spectrum member of the tetracycline family of antibiotics (Figure 1), which is used for treating several bacterial infections through the inhibition of bacterial protein synthesis [114]. In addition to this activity, doxycycline showed inhibitory effects on angiogenesis and matrix metalloproteinases (MMPs) activity, thus, these properties led to the exploration of the potential impact of the drug in several malignancies [115]. In pancreatic cancer, doxycycline showed to affect PC growth by activating proapoptotic genes and suppressing antiapoptotic genes, perturbating cell cycle and inhibiting the expression of the proangiogenic IL-8 [34,35]. Doxycycline also reduced tumor growth by 80% in a xenograft mouse model of PC [34]. Considering that mitochondrial ribosomes are evolutionarily related to bacterial ribosomes, it is well known that several classes of antibiotics, including tetracycline, target mitochondria as a side effect, interfering with mitochondrial biogenesis and function [36,116]. Using an unbiased proteomic approach, Lamb et al. showed the upregulation of several mitochondrial-related proteins in mammospheres, as compared with monolayers, supporting the hypothesis that tumorsphere formation is dependent on mitochondrial biogenesis [117]. Based on these considerations, the authors explored the capacity of different antibiotics targeting mitochondria, including doxycycline, in inhibiting tumorsphere formation in multiple cancer cell lines [36]. Specifically, doxycycline reduced tumorsphere formation in PC cells at a concentration that did not affect the viability of both bulk cancer cells and normal fibroblasts, showing its potential in selectively targeting tumor-initiating cancer stem cells reponsible for tumor recurrence, metastasis and drug resistance. Similarly, Dijk and co-workers showed that inhibition of mitochondrial protein synthesis by doxycline, combined with gemcitabine, decreases PC cell proliferation via ATP depletion. Moreover, the combined treatment promotes gemcitabine-induced apoptosis by decreasing mitochondrial membrane potential and fostering ROS production [37]. Recently, Liu et al. explored both in vitro and in vivo the effects of doxycline, as a protease-activated receptor 1 (PAR1) inhibitor, on PC cancer stem cell properties [38]. In particular, the authors show that PAR1 promotes cancer stem cell features and EMT of PC cells. In this context, doxycline affects PC growth, migration, invasion and tumorsphere formation through the down-regulation of PAR1/FAK/PI3K/AKT signaling pathway. Moreover, when used in combination with different chemotherapeutic drugs, doxycyclin displays synergistic effects on the viability of PC cells, with a particular enhancement of 5-FU effects. These findings were confirmed also in vivo, where the combination of doxycycline and 5-FU inhibited PC cell growth by 80.5% in subcutaneous Panc-1 xenografts models, as compared with single agents, together with the increase in E-Cadherin expression and the decrease in Vimentin and CD133 expression [38].

There are no published data on clinical trials of doxycyline in PC. As an anti-cancer agent, it was tested in a phase II trial in combination with interferon-alpha in advanced renal cell carcinoma, with disappointing results [118]. However, this drug is being tested in a window-of-opportunity trial in patients with resectable pancreatic cancer who will receive neoadjuvant chemoradiation therapy. The aim of the study is to assess the stem-cell cytocidal effects of 8 weeks treatment with doxycycline 100 mg twice daily, administered prior to surgery (NCT02775695).

### 2.6. HIV Inhibitors (Efavirenz, Nelfinavir, Ritonavir)

Anti-viral drugs are commonly employed for the treatment of a wide range of human infectious viral diseases, including hepatitis, influenza, cytomegalovirus and also immune deficiency virus (HIV) [119]. Among others, HIV inhibitors efavirenz, nelfinavir and ritonavir gained a particular attention because of relevant anticancer effects displayed in a large variety of tumors, including pancreatic cancer [120,121]. In particular, efavirenz is an anti-viral drug based on the 1,4-dihydro-2H-3,1-benzoxazin-2-one scaffold (Figure 1) and has good long-term tolerability in HIV-1-infected patients [122]. It hampered clonogenicity and induced apoptosis in distinct PC cell lines, where the drug affected viability with IC_50_ values comparable with those achieved by the drug when used in distinct cohort of HIV patients [39]. In addition, a radio-sensitizing effect of efavirenz was reported in PC cells, where the combined treatment of the drug with radiation enhanced its antiproliferative effect, through a mechanism involving ROS production and mitochondrial membrane depolarization, along with phosphorylation of both ERK1/2 and p38 MAPK stress pathways in cancer cells [40]. There are no studies of efavirenz in animal models of PC, whereas the drug showed in vivo antitumor effects in other cancer models, including human thyroid anaplastic and lung squamous cell carcinomas [123,124]. As far as the peptidomimetic nelfinavir (Figure 1), a study explored its antitumor potential in epidermal growth factor receptor/HER2 overexpressing PC cell lines [41]. In fact, constitutive activation of K-ras, a common feature in PC, confers radio-resistance also through the activation of PI3K/Akt and Raf/MEK/ERK downstream signaling pathways [125,126]. In this regard, Kimple et al. investigated the role of nelfinavir as a radiosensitizer in PC due to the ability of this and other HIV-protease inhibitors to block Akt signaling, thereby radiosensitizing cancer cells both in vitro and in vivo [41,127]. Nelfinavir, at a clinically achievable dose, showed in vitro efficacy by inhibiting Akt phosphorylation in PC cell lines, which in turn radiosensitized both wild-type and K-ras mutant cell lines. Consistently, phospho-Akt levels were significantly decreased also in Capan-2-bearing xenografts treated with a combination of clinically relevant doses of radiation and oral nelfinavir, which synergistically delayed tumor growth in mice [41]. A further study by Veschi et al. explored the antitumor potential of nelfinavir as single drug, or in combination in several PC cell lines with distinct genetic profiles [42]. The drug impaired clonogenicity and decreased viability by affecting cell cycle and promoting apoptosis in PC cells. Interestingly, these antitumor effects were enhanced and synergistic when nelfinavir was used in combination with nitroxoline, another repurposed drug candidate in PC (see below), regardless of the presence of erlotinib, a targeted agent already approved in PC treatment [42]. Although nelfinavir appears a promising candidate drug to be repurposed in PC, a main issue associated with this drug still remains [19]. In fact, at present nelfinavir is hardly used for the original indication, thus, it is no longer marketed in Europe and this aspect may complicate its supply for clinical trials [19]. Ritonavir is another peptidomimetic HIV-protease inhibitor (Figure 1) effective in HIV patients, with relatively few side effects, which has been shown to have antitumor properties in PC. Specifically, Batchu et al. reported that ritonavir affects PC cell viability by triggering the intrinsic apoptotic pathway and interfering with cell cycle machinery via suppression of Akt and Rb phosphorylation in these cancer cells [43]. Moreover, the drug hampers PC cell motility and invasiveness. Remarkably, ritonavir has a greater antiproliferative effect in PC cells when used in combination with gemcitabine at clinically relevant, nontoxic doses, thus supporting its potential as repurposed drug candidate in PC [43]. Similarly to efavirenz, ritonavir has not been tested in animal models of PC. However, the drug proved to be effective in reducing growth in other tumor models as a single agent, or in combination [128,129,130].

Among the aforementioned HIV inhibitors, nelfinavir has been tested in borderline resectable, or locally advanced PC in association with either conventionally fractionated, or stereotactic radiation therapy, due to the relative radio-resistance of PC. Generally, phase I trials have found the association to be well tolerated at the standard dose of antiretroviral treatment (1250 mg bid), and most of the observed toxicities were likely not related to nelfinavir [82,83]. Specifically, the maximum tolerated dose (MTD) of nelfinavir as an anticancer agent was determined for different cancers, including PC, and found to be of 2.5 times higher than the dose approved for HIV management [131]. Nelfinavir has been also investigated in at least two phase II trials [84,85], both prematurely terminated due to drug unavailability or suboptimal treatment. Although indirect comparisons with historical controls indicate a potential increase in the rate of response to treatment, none of the published studies allows a clear assessment of the additive benefit of the drug. However, in one study, response was associated to post-nelfinavir changes in tumor hypoxia and perfusion, as evaluated by functional imaging [84]. Major insights on the utility of nelfinavir will probably come from the SCALOP-2 study, in which the first dose-finding stage has been completed and the MTD for nelfinavir established at 1250 mg [86,132]. In the second stage of the study, patients will be randomized to receive three additional cycles of gemcitabine/nab-Paclitaxel, capecitabine with standard or high dose radiotherapy, with or without nelfinavir at 1250 mg bid [86].

### 2.7. Hydroxychloroquine and Chloroquine

Both chloroquine and hydroxychloroquine are 4-aminoquinolines (Figure 1) approved for the treatment of malaria and autoimmune disorders, including lupus and rheumatoid arthritis [133]. These drugs have been shown to display antitumor properties in several malignancies by affecting both cancer cells and components of tumor microenvironment [134,135]. Their antitumor effects are mainly attributable to the inhibition of autophagy, which in turn prevents degradation of metabolic stress products and thus promotes cell apoptosis [134,136]. Autophagy is a complex process that allows cancer cells to recycle bioenergetic metabolic stress products generated by increased metabolic activity and hypoxia associated with higher cell turnover within solid tumors, in order to maintain energy homeostasis and cell survival [137]. Autophagy has been shown to be upregulated, especially in the later stages of pancreatic intraductal neoplasia (PanIN) progression to PC, and appears to be implicated in resistance to both chemotherapy and targeted therapies [44,138]. Yang et al. first proved that chloroquine decreased proliferation in several PC cell lines and markedly improved the survival of murine xenograft models of pancreatic cancer [44]. Similarly, Endo et al. showed that inhibition of autophagy by chloroquine promoted a quiescent state of pancreatic stellate cells (PSCs), reduced expression of the invasive cytokine IL-6 and production of extracellular matrix proteins, thereby attenuating invasion properties and liver metastasis formation in an orthotopic PC mouse model [45]. In addition, Frieboes et al. showed that chloroquine affected viability of metastatic PC cell lines, and its effects were enhanced in hypoxic conditions [46]. However, although promising preclinical results encouraged clinical evaluation of chloroquine in PC treatment, subsequent clinical trials reported limited to no efficacy of chloroquine as monotherapy [139]. Thus, several studies explored the effects of combining chloroquine with other drugs acting on different molecular targets and pathways. In this regard, considering that chemotherapy boosts autophagy in cancer cells, which in turn promotes cell survival, as a mechanism of resistance to therapy [138], it is conceivable that standard chemotherapy combined with a treatment aimed at inhibiting autophagy may reverse that resistance. Consistently, Hashimoto and colleagues showed that autophagy is strongly induced as a cytoprotective mechanism against 5-fluorouracil, or gemcitabine treatments in PANC-1 and BxPC-3 pancreatic cancer cell lines [47]. Remarkably, the combination of chloroquine with 5-fluorouracil or gemcitabine augmented the antiproliferative effects of the two chemotherapy drugs in those PC cell lines [47]. Interestingly, Fu at al. demonstrated that chloroquine cooperated with gemcitabine in reducing growth in xenograft pancreatic cancer models, whereas chloroquine alone, differently from the previous study by Yang et al. [44], was ineffective [48]. Moreover, inhibition of autophagy by chloroquine in PC cells was more effective than the ATG7 siRNA strategy in increasing gemcitabine-induced cytotoxicity and chloroquine pretreatment, followed by gemcitabine, markedly triggering reactive oxygen species (ROS) release and thus stimulating lysosomal membrane permeabilization and subsequent apoptosis [48]. Overall, these data suggest a role of chloroquine as a gemcitabine chemotherapy sensitizer in PC treatment.

In addition to gemcitabine, chloroquine proved to be effective in combination with other drugs in different preclinical PC models. Noteworthy, the combined treatment with ERK/MAPK inhibitors and chloroquine synergistically enhanced chloroquine-mediated growth inhibition in both organoid and patient-derived xenografted (PDX) models of PC [49]. Specifically, inhibition of ERK/MAPK signaling impaired mitochondrial functions and glycolytic activity in PC cells, which in turn both generated a state of autophagy dependence and increased sensitivity to autophagy inhibition by chloroquine [49]. Both in vitro and in vivo models indicate that autophagy inhibition by chloroquine hampers immune escape of PC cells [50].

An additional proposed mechanism distinct from autophagy, which has been described for explaining the antitumor activity of chloroquine, is related at least in part to the inhibition of both CXCL12/CXCR4 and Hedgehog signaling pathways in pancreatic CSCc (Pa-CSCs) [51]. In particular, Balic et al. showed that chloroquine preferentially eliminates Pa-CSCs in primary PC cell cultures by promoting internalization of C-X-C chemokine receptor type 4 (CXCR4) in CSCs, thereby decreasing cell sensitivity to chemokine (C-X-C motif) ligand 12 (CXCL12). Inhibition of CXCL12/CXCR4 signaling by chloroquine resulted in reduced phosphorylation of the extracellular signal-regulated kinase (ERK) and the signal transducer and activator of transcription 3 (STAT3), as well as in the downregulation of Hedgehog signaling. The latter pathway is quiescent in physiological conditions, but its activation is involved in tumorigenesis and in maintaining a stem-like aggressive phenotype. In vivo, chloroquine-treated cells, either alone or in combination with gemcitabine, showed drastically reduced tumorigenicity and invasiveness in a panel of pancreatic cancer PDX models, also improving outcomes of mice bearing primary xenografts [51]. Thus, this study supports the use of chloroquine as an inhibitor of Pa-CSCs, which may be combined with gemcitabine-based approaches that target the tumor bulk.

The combination of gemcitabine with chloroquine as first or late-line treatment in patients with unresectable/metastatic cancer has been assessed in a phase I study and found to be safe and well tolerated [78]. Similarly, safety and tolerability of hydroxychloroquine in combination with gemcitabine have been evaluated in a phase I/II trial (NCT01128296). Thirty-five patients with resectable pancreatic cancer were preoperatively treated with two infusion of fixed-dose rate gemcitabine and escalating doses of daily hydroxychloroquine for 31 consecutive days [79]. Treatment was safe and tolerable, with no dose-limiting toxicity or treatment delays. Notably, in this study, LC3-II levels in peripheral lymphocytes were used as a surrogate marker of autophagy inhibition. Interestingly, patients with increased levels of LC3-II showed improved PFS and OS. Rate of resection and R0 resection were reported to be higher than those observed in prior series from the same institution [79,140]. However, these results should be considered with caution, given the limited size of the study and the indirect nature of the comparison.

Hydroxychloroquine was also evaluated in a randomized phase II trial in which 98 patients with resectable or borderline resectable tumors received two cycles of gemcitabine/nab-paclitaxel, either alone or in combination with the drug (NCT01978184). The observed attrition rate was 36%, with no significant difference between the arms. The rate of grade IIB or higher histopathologic response was significantly higher in the combination arm (55.9% versus 10%) and was correlated with the decrease in Ca19.9 serum levels. Treatment with hydroxychloroquine was also associated with an increased staining of tumor specimens for SQSTM1/p62, suggesting a reduced autophagy, and with a higher tumor infiltration by immune cells. Both reduced autophagy and higher tumor infiltration by immune cells are in line with a beneficial role of the drug in reverting tumor immunoescape mechanisms observed in vitro and in animal models [50]. No differences in OS or RFS were observed [80]. In the advanced/metastatic setting, a randomized phase II study explored the contribution of hydroxychloroquine when used in combination with first line gemcitabine/nab-paclitaxel (NCT01506973). Although the trial failed to show an improvement in 1-year OS, the overall response rate was significantly higher for patients receiving hydroxychloroquine (38.2% vs. 21.1%), with a manageable increase in toxicity [81]. This, together with the increased histopathological response observed by Zeh et al. [80], suggests that the ideal setting for the combination of these agents with chemotherapy would be the preoperative one, where an increased response may imply a higher resection rate [81,138].

Preclinical data consistently indicate that inhibition of the RAS-RAF-MEK-ERK pathway results in an increased autophagy dependence in cancers driven by KRAS or BRAF mutations (90% of pancreatic cancer) [49,141]. Moreover, it has been shown that chloroquine or hydroxychloroquine synergize with inhibitors of RAF (binimetinib), MEK (trametinib) or ERK (SCH772984, ulixertinib) in suppressing tumor growth [49,141]. Kinsey et al. also reported the case of a heavily pretreated patient with pancreatic cancer that was treated with compassionate trametinib and hydroxychloroquine. The patient achieved a partial response according to RECIST criteria after 4 months of treatment [141]. Based on this evidence, at least two phase I and one phase I/II clinical trials have been activated to assess the combination of hydroxychloroquine with either binimetinib (NCT04132505), trametinib (NCT03825289) or the ERK inhibitor LY3214996 (NCT04386057).

### 2.8. Itraconazole

Itraconazole is a synthetic broad-spectrum anti-fungal triazole drug (Figure 1), which acts through the deregulation of the ergosterol biosynthesis pathway in fungi [142]. This drug is used as an orally systemic and relatively safe treatment that can be continued for months, with rare side effects [142]. Interestingly, its antineoplastic activity is well documented by a large number of in vivo, in vitro and clinical evidence in several tumors, either as a single agent or in combination with standard chemotherapy drugs [143]. Multiple underlying mechanisms have been proposed to explain the promising antitumor properties of itraconzonale, including inhibition of endothelial cell proliferation, prevention of angiogenesis, induction of autophagic growth arrest and reversal of drug resistance through the inhibition of the P-glycoprotein drug efflux pump [143]. In addition, itraconazole has been shown to affect the Hedgehog signaling pathway, which is known to be abnormally modulated in pancreatic cancer, although with contradictory results indicating either a promoting or limiting role of this signaling pathway in PC progression [144,145,146]. Indeed, to date, there are few preclinical studies and no clinical trials investigating antitumor effects of itraconazole in PC [147]. Specifically, Jiang et al. reported that this drug inhibits PC cell growth in vitro by inducing apoptosis through ROS release, which in turn promotes mitochondrial membrane depolarization and Bak-1 activation [52]. Itraconazole was also shown to be effective in vivo, significantly inhibiting pancreatic cancer xenograft tumor growth, with no toxicity for the animals [52]. Similarly, Chen et al. showed that itraconazole inhibits PC cell viability by inducing apoptosis and impairs migration, invasion and epithelial to mesenchymal transition (EMT) of PC cells at least in part through the suppression of TGF-β/SMAD2/3 signaling [53]. Furthermore, itraconazole was able to suppress tumor growth in a genetically engineered mouse model (GEMM) of PC that spontaneously develops pancreatic cancer [53]. Considering that itraconazole appears to modulate multiple pathways essential in malignant progression, further studies will be warranted to corroborate the therapeutic value of itraconazole as repurposed drug candidate in PC treatment.

The antitumor effect of itraconazole has been assessed in a retrospective mono-institutional cohort of patients with metastatic PC progressing after first or second-line treatments [148]. They were treated with a three-drug combination (docetaxel, gemcitabine and carboplatin) in combination with itraconazole with an overall response rate (ORR) of 37%. Over 90% of patients were subsequently treated with irinotecan-containing regimens and itraconazole, with a final ORR of 47%. Toxicity was limited, with the exception of transfusion-requiring anemia, which was observed in 61% of patients. Median OS was of 11.4 months, which substantially exceeded that of 6 months reported in a comprehensive analysis of published second-line trials [149]. The authors suggested that the improved outcome might be related to the anticancer effect of itraconazole, although they acknowledged that the retrospective nature of the study does not rule out the possibility that this was dependent on the use of a three-drug combination [148].

### 2.9. Losartan

Losartan is a selective, non-peptidic angiotensin II type 1 (AT1) receptor blocker (ARB) (Figure 1) that is widely used as a relatively safe drug to treat hypertension [150]. In general, the renin–angiotensin–aldosterone system (RAAS) and its key components, including the bioactive angiotensin II and its AT1 and angiotensin II type 2 (AT2) receptor subtypes, are well-recognized regulators of blood pressure homeostasis and electrolyte balance. The detection of local RAAS signaling in various organs and tissues pointed out its relevance in cell biological processes, as well as in pathophysiological processes, such as inflammation [151]. Remarkably, there is a large body of evidence showing that angiotensin II also acts as a paracrine and/or autocrine signal in the tumor microenvironment, thereby promoting the recruitment of inflammatory cells. This, in turn, leads to an increased secretion of tumor growth factors that boost both cell proliferation and growth of stromal cells, as well as tumor angiogenesis by up-regulating vascular endothelial growth factor (VEGF) expression [151,152,153,154]. Based on these considerations, losartan was extensively tested in different preclinical tumor models, including pancreatic cancer, for its anti-angiogenic, anti-fibrotic and thus anti-neoplastic potential [155,156,157,158]. In particular, a study pointed out an improved survival benefit in rat orthotopic pancreatic cancer models treated with a combination of losartan and gemcitabine through a mechanism involving the inhibition of VEGF synthesis and suppression of PC cell proliferation via AT1R blockade [54]. Similarly, Li et al. reported that the combined therapy based on the sequential administration of losartan- followed by gemcitabine-loaded magnetic mesoporous organosilica spheres significantly inhibits PC growth in vivo, as compared to monotherapy strategy, with negligible toxicity in mice, through the reduction in major solid components in the extracellular matrix (ECM) of the tumor, which in turn facilitates the penetration of nanodrugs into the tumor site [55]. Moreover, Chauhan et al. showed that losartan is able to impair stromal collagen and hyaluronan production through the decrease in profibrotic signal expression released by cancer-associated fibroblasts (CAFs) in desmoplastic tumours, such as pancreatic cancer [56]. This effect promotes tumor vessel decompression and significantly improves tumor perfusion, which in turn enhance oxygen, chemo and nanotherapeutics delivery, fostering overall survival in orthotopic PC mouse models [56,57]. It should be noted that these effects were consistently observed in the presence of distinct ARBs, suggesting that their antitumor activities are mediated by AT1R inhibition rather than by off-target effects [56]. Interestingly, the same authors reported that, both in mouse models of PC and in patients, obesity promotes tumor desmoplasia and PC growth by a complex cross-talk among adipocytes, tumor-associated neutrophils (TANs) recruited by adipocyte-secreted IL1β and pancreatic stellate cells (PSCs) [58]. In such a scenario, the AT1R blockade by losartan was shown to attenuate obesity-induced fibrosis and tumor progression, and to improve response to chemotherapy. Moreover, this blockade also has effects on immune cells in PC microenvironment, suggesting a beneficial role in reversing tumor immunoescape [58,59]. It is worth noting that ablation of CAFs, or more in general stromal-targeting approaches for PC, yielded contradictory results, underlining how stromal components may promote or hamper cancer progression [155,159]. In this regard, losartan as an anti-fibrotic agent to be used in combination with conventional chemoradiotherapy, but possibly also with immunotherapy and/or antiangiogenic therapy, warrants particular attention because it seems not to increase tumor metastasis risk [155].

The effect of losartan in improving chemotherapy delivery to cancer cells has been explored as a secondary endpoint in a clinical trial of intraoperative gemcitabine associated, or not to 1 to 3 week oral losartan, aimed at correlating incorporation of gemcitabine into DNA with transport properties of PC tissue derived from contrast-enhanced CT scan analysis (NCT01276613). However, results have been provided for the gemcitabine arm only [160]. Liu et al. retrospectively correlated chronic use of angiotensin inhibitors (AI) with survival in a cohort of 794 consecutive patients with PC [161]. Multivariate and propensity score analysis suggested that assumption of AI is associated with significantly longer survival in non-metastatic patients independently from adjuvant chemotherapy, likely due to stroma normalization and stimulation of antitumor immune response [161]. This evidence formed the background for a phase II clinical trial in which 49 patients with locally advanced pancreatic cancer were treated with eight cycles of neoadjuvant FOLFIRINOX combined with losartan and followed by chemoradiotherapy (CRT) [87]. The primary endpoint of the study was to assess the rate of resection with negative margins (R0). The non-randomized design and the limited size of the study do not allow conclusions about losartan contribution, but the observed R0 resection rate of 61% is encouraging [87]. More insights on the role of losartan will likely come from a multi-institutional phase II study in which patients with resectable or borderline resectable pancreatic cancer will be initially treated with four cycles of FOLFIRINOX. In case of response or no disease progression, they will receive four additional courses, or will be be switched to gemcitabine/nab-paclitaxel in case of toxicity or progression. Finally, patients will be treated with short or long-course chemoradiation before surgery. Losartan will be administered for the entire duration of the treatment (NCT04539808).

### 2.10. Metformin

The anticancer properties of this biguanide compound (Figure 1) have been extensively investigated in preclinical models of PC, and attributed to both direct and indirect actions. Among others, direct effects on cancer cells may be due to the disruption of mitochondrial oxidative phosphorylation through inhibition of respiratory complex I, which reduces ATP production [162]. This, in turn, leads to an increase in the AMP/ATP ratio and to the activation of AMPK signaling that negatively regulates mammalian target of rapamycin (mTOR) through phosphorylation of the tuberous sclerosis complex 2 (TSC2), which induces the reduction in protein synthesis and cell proliferation [60,163,164,165]. Metformin may also indirectly affect PC cell growth. In particular, by decreasing hepatic energy state, it reduces endogenous glucose production via gluconeogenesis [166]. As a consequence, glucose blood levels decrease and this secondarily lowers insulin levels and reduces insulin/insulin-like growth-factor-1 signaling. This attenuates both downstream signaling through PI3K/Akt and through Ras/Raf/mitogen activated protein (MAP) kinase pathways [61,62,167,168]. However, in contrast with these effects on signaling pathways, it should be noted that metformin was not able to inhibit PC growth in a mouse model of patient-derived xenograft [169]. On the other hand, it was shown that the antitumor effect of metformin may be also mediated by direct effect on CD8+ T-cells [170] and by inhibition of oncogenic micro-RNAs [171]. Intriguingly, metformin was shown to hamper PD-L1/PD-1 mediated immunoescape in different preclinical cancer models [172].

This evidence and the high prevalence of diabetes among patients with pancreatic cancer [173] justified the assessment of metformin as an adjuvant to standard chemotherapy in these patients. In the past decade, several retrospective cohort studies assessing the effect of metformin in diabetic patients with pancreatic cancer have been published, with mixed results [174,175,176,177,178,179,180,181,182]. However, these studies suggested an overall positive effect of metformin on PC patient survival, as demonstrated by different meta-analyses [183,184,185].

On the contrary, three phase II trials, two of which were randomized, all enrolling PC patients not assuming metformin, or non-diabetic, demonstrated no beneficial effects of metformin. Braghiroli et al. conducted a phase II, Simon’s two-stage trial of metformin and paclitaxel for patients with gemcitabine refractory advanced pancreatic cancer. The study failed to achieve the hypothesized disease control rate (DCR) after enrolling the first 20 patients and, therefore, was terminated for futility [88]. In a phase II randomized, placebo-controlled trial, Kordes and colleagues assessed the effect of metformin when added to gemcitabine and erlotinib as first- or second-line therapy for locally advanced/metastatic disease [89]. This treatment backbone was chosen because of preclinical evidence of a synergistic effect of metformin and inhibitors of EGFR-tyrosine kinase [186,187]. Moreover, plasma metformin concentrations reached in the study were similar to plasma concentrations in diabetes patients treated with the drug (approximately 7 µmol/L). The study was powered to detect a 50% increase in 6-month OS and required the enrollment of at least 120 patients. There was no significant difference in 6-month OS between the two treatment groups (placebo arm: 63.9%; metformin arm: 56.7%). Although the incidence of grade 3–4 adverse events was similar between groups, doses of metformin (1000 mg twice a day) were reduced more often than placebo and the median number of cycles received was lower (three vs. five). As higher plasma levels of metformin were associated with an improved survival, the authors argued that efficient intracellular concentration of metformin is not obtainable at the anti-diabetic dose used in the study [89]. Indeed, it should be noted that most in vitro models showing antineoplastic effects of metformin employed supra physiological drug concentrations, in the range of mmol/L, whereas plasma concentrations in diabetes patients usually are in the range of µmol/L.

The other randomized phase II trial was an open-label study testing the efficacy of metformin associated to a four-drug regimen including cisplatin, epirubicin, capecitabine and gemcitabine (PEXG) [90] in patients with metastatic disease. The primary endpoint of the study was to assess a 20% absolute increase (from 50% to 70%) of 6-month PFS. At the preplanned interim analysis, after 60 of the planned 78 patients were enrolled, no difference in 6-month PFS between the metformin and the control arm (42% vs. 52%) was observed. Median OS was 10.4 months and 6.8 months, while the DCR was 79.5% and 64.5% in the control and the metformin group, respectively. Multivariate Cox analysis PFS indicated a significantly increased risk of progression for the metformin arm (HR = 2). Therefore, the study was ended for futility. The observed trend towards a worse prognosis for patients receiving metformin could be related to the ability of this drug to reduce the generation of reactive oxygen species, which may have antagonized the effects of chemotherapy [188].

Although methodological and clinical limitations of the reported randomized trials have been advocated as factors masking potential benefits of metformin [189], intrinsic limits of retrospective observational studies cannot be ignored. Among them, heterogeneity of the cohorts in terms of cancer stage, pre-existence of the diabetic status and misclassification of metformin use have been suggested. Time-related biases, in particular, immortal time bias [190], may lead to an overestimation of metformin effect [178,191]. Indeed, when time of metformin assumption was accounted for, results of observational studies [178,180,182] and meta-analyses [192,193] suggest that the advantage of metformin might be only apparent. However, methodological and clinical limitations of the reported randomized trials have been advocated as factors potentially masking benefits of metformin [189].

### 2.11. Nitroxoline

Nitroxoline is an old antibiotic, belonging to the class of 8-hydroxyquinolines (Figure 1), used for the treatment of urinary tract infections, that received attention for its antitumor activities displayed in preclinical cancer models, including xenograft and genetically modified mice models of several tumors [14,194,195,196,197]. Recently, nitroxoline proved to be effective also in PC [42,63,198]. The drug was able to affect viability of different PC cell lines, by altering cell cycle and fostering apoptosis. Moreover, it markedly hampered self-renewal capacity, migration and invasion of PC cells, although with slight differences in potency among the three tested PC cell lines possibly due to their distinct genetic backgrounds [42,63,198]. Remarkably, combinations of nitroxoline with the HIV-protease inhibitor nelfinavir, with or without erlotinib, resulted in dose-dependent synergistic effects on PC cell viability, paralleled by profound cell cycle perturbation, drastic clonogenicity impairment and more consistent apoptosis promotion, as compared to single agents [42]. To unravel possible mechanisms of nitroxoline anticancer activity in PC cells, an integrative approach based on proteomic and functional analyses was employed [63]. Nitroxoline was revealed to affect multiple crucial biological pathways and oncogenic proteins in PC, previously known or unknown to play a role for explaining anticancer activity of the drug [14,63,194]. Specifically, nitroxoline promoted ROS production and induced DNA damage response through a mechanism involving the deregulation of Na/K ATPase pump and β-catenin pathway. Moreover, the drug fostered the deregulation of molecules involved in cell bioenergetics, thereby leading to mitochondrial depolarization, together with the impairment of cytosolic iron homeostasis [63].

There are no clinical studies of nitroxoline in PC. However, nitroxoline was approved for a phase II clinical trial in China designed to the treatment of non-muscle invasive bladder cancer (CTR20131716), and the results of this trial were not published yet [196].

### 2.12. Pimavanserin

Pimavanserin is an atypical urea-based antipsychotic drug (Figure 1) newly approved for the treatment of Parkinson’s disease psychosis [199]. Recently, Ramachandran and Srivastava reported promising in vitro and in vivo antitumor effects of pimavanserin in PC [64]. The drug affected viability and clonogenicity of multiple PC cells with distinct sensitivities to gemcitabine, showing negligible effects on normal pancreatic epithelial cells and lung fibroblasts. These antiproliferative effects, together with the induction of apoptosis, were mediated by the inhibition of the Akt/Gli1 signaling cascade, a well recognized oncogenic axis in pancreatic cancer [200,201]. Moreover, pimavanserin also impaired the expression of several Gli1 downstream cancer stem cell markers, including Oct-4, SOX2 and NANOG, and suppressed the size and number of PANC1 tumorspheres. In vivo, oral administration of pimavanserin markedly reduced tumor growth in both subcutaneous and orthotopic xenografts, without any general signs of toxicity, or behavioral changes in mice, thus supporting the potential of this drug as a candidate to be repurposed in PC [64].

No clinical trials of pimavanserin in PC were reported so far, and overall, there is a lack of clinical studies regarding this drug in cancer.

### 2.13. Pirfenidone

Pirfenidone is an orally active and synthetic 2-pyridinone (Figure 1) approved for the treatment of idiopathic pulmonary fibrosis [202]. Notably, this drug proved to be effective in inhibiting fibrosis and/or desmoplasia, as well as fibroblast proliferation by affecting TGF-beta, PDGF and collagen type I production in several in vitro and in vivo models of lung and hepatic fibrosis [203,204,205]. Considering its antifibrotic effects, pirfenidone was also tested in epithelial cancers frequently displaying desmoplasia, including PC [206,207]. Desmoplasia is due to an abnormal activation of pancreatic stellate cells (PSCs), that in normal conditions constitute 4–7% of the total cell population of pancreas. The histologic features of desmoplasia include overproduction of extracellular matrix, extensive proliferation of PSCs and transformation into myofibroblast-like cells expressing α-smooth muscle actin (SMA), few immune cells, endothelial cells, nerves and matrix proteins. Desmoplasia promotes cancer progression and reduces effective drug delivery.

Pirfenidone was shown to suppress cell proliferation and induce G0/G1 cell cycle arrest in PSCs and in different human PC cells [65]. Expression of p21 was increased in pirfenidone-treated PCs, but not that of CDK2, and there was no significant apoptosis [65]. Another study consistently showed that pirfenidone exerted dose-dependent antiproliferative effects on PSCs and PC cells, but it had stronger inhibitory effects on PSCs than PC cells when used at low dosages [66]. Moreover, the drug significantly decreased invasion and migration of PSCs. Interestingly, pirfenidone-treated PSCs did not enhance the proliferation, invasiveness and migration of PC cells due to the suppression of platelet-derived growth factor-A (PDGF-A), hepatic growth factor (HGF), periostin, collagen type I and fibronectin production in these cells [66]. In vivo, pirfenidone was able to suppress PC growth and metastasis by disrupting tumor-stromal interactions [66]. In particular, periostin, or osteoblast-specific factor 2, regulates collagen fibrillation, activates Wnt signaling, supports cancer stem cells and metastasis [208]. This protein is overexpressed in PC and is associated to a poorer prognosis [66]. Mice co-implanted with SUIT-2 pancreatic cancer cells and PSCs showed marked desmoplasia, with larger Sirius red-positive areas and more α-SMA-positive cells than the implants of SUIT-2 cells alone. Pirfenidone significantly reduced the growth, the PCNA index, the Sirius red-positive areas and α-SMA-positive cells in tumors consisting of SUIT-2 cells co-implanted with PSCs, but not in tumors consisting of SUIT-2 cells alone [66]. An additional study reported that pirfenidone inhibited desmoplastic reactions and tumor growth in HapT1-derived orthotopic hamster PC models, reduced the number of α-SMA-positive cells, collagen deposition and the number of proliferating cells (Ki67 reduction) [67]. Extracellular vesicles (EV) released by anti-inflammatory tumor-associated macrophages (TAMs) reduce PC sensitivity to gemcitabine through activation of ERK and beta-catenin [209]. Chitinase 3-like-1 (CHI3L1) and fibronectin (FN1) were detected among the most abundant proteins of macrophages-derived EVs [68]. In particular, they were found in the stroma of PC samples associated with a high presence of macrophages, drug resistance and reduced patient survival. Pirfenidone inhibited CHI3L1 and FN1 in PC cells, partially reverting their resistance to gemcitabine [68]. β-cyclodextrin (β-CD)-modified matrix metalloproteinase-2 (MMP-2) responsive liposomes integrating pirfenidone and gemcitabine (LRC-GEM-PFD) and matrix metalloproteinase-2 (MMP-2) responsive peptide-hybrid liposome (MRPL)–pirfenidone complexes were synthetized to allow better penetration of the drugs into PC microenvironment and improved perfusion of gemcitabine. LRC-GEM-PFD down-regulated collagen I and TGF-β in PSCs, reduced stromal fibrosis, increased drug perfusion and reduced tumor growth [69,70]. In addition, pirfenidone/gemcitabine hollow mesoporous organosilica nanoparticles (HMON) complexes showed good penetration and improved gemcitabine release and cytotoxicity by fostering apoptosis of SW1990 PC cells and reducing collagen I and fibronectin components of the ECM [71]. In vivo, the complex downregulated collagen I and fibronectin components of the ECM, increased apoptosis and endovascular osmotic pressure, reduced tumor growth and prolonged survival in SW1990/PSCs tumor bearing mice [71].

No clinical trial of pirfenidone in PC patients has been reported. Of note, two phase II trials in patients with neurofibromatosis type 1 have been completed and showed only modest activity [210,211].

### 2.14. Propranolol

Both sensitive and autonomic nerve systems innervate the pancreas and act as pivotal players in the development and progression of PC by modulating the immune system, stress response and by guiding neural invasion [212,213,214]. A common feature in PC is the “perineural invasion”, which is driven by neurons and is associated with “neurogenesis”, an abundant infiltration of the tumor by the autonomic nervous system, high neural density, marked neural hypertrophy and overexpression of norepinephrine [215,216]. Notably, mice with PC show a 30-fold increase in nerve area when compared with normal pancreas [72]. In PC patients, 71–100% show intra- and extra-pancreatic perineural invasion by cancer cells, which has a major impact on patient survival [217]. The local microenvironment of PC and tumor cells also stimulate nerve growth by secreting neurotrophins (NTs), including nerve growth factor (NGF) and brain-derived neurotrophic factor (BDNF), mediate axonal guidance and synaptic plasticity, protect nerves from injury and potentiate tumor cell invasion [216,218]. In particular, a prevalence of the sympathetic system, higher catecholamine levels, or a reduced parasympathetic activity, stimulate the growth and progression of PC [219]. On the other hand, reduced sympathetic activity or an increased parasympathetic tone are protective [219]. Notably, both chronic and acute stress induce sympathetic system activation. In this regard, PC patients have psychological stress levels that result to be the highest among all types of cancers [220]. The stress response is modulated through the release of endogenous catecholamines (norepinephrine and epinephrine) and the upregulation of B-adrenoceptors (B1 and B2), which are members of the superfamily of G protein-coupled receptors.

In such a scenario, the non-selective B2/B1-adrenergic antagonist propranolol (Figure 1) was shown to have a beneficial effect in preclinical models of PC and also in patients [72,73,74,75]. It should be noted that in principle, non-selective beta-blockers (BBs) might have a greater effect on inhibiting cancer progression due to their ability to hamper both the cAMP/PKA and Ras pathways, as compared to selective BBs that only inhibit the cAMP/PKA pathway [73,74]. Zhang et al. reported that propranolol is able to suppress PC cell proliferation and invasiveness in vitro and in vivo by inducing apoptosis and inhibiting the expression of factors responsible for invasion, including nuclear factor κB (NFκB), activator protein 1 (AP-1) and cAMP response element binding protein (CREB), as well as the expression of MMP-9, MMP-2 and vascular endothelial growth factor (VEGF) target genes [73,74]. Interestingly, Partecke and colleagues demonstrated the relevant impact of chronic stress and activated β-adrenergic receptor signaling in a clinically relevant immunocompetent orthotopic syngeneic murine model of PC [75]. Chronically stressed mice showed increased tumor growth and reduced survival through the enhancement of MMP2/MMP9 expression, the induction of stress markers steroids and adrenal tyrosine hydroxylase, a key enzyme in the production of catecholamines, and the suppression of immune response, with a trend toward fewer CD4 and increased intratumoral Tregs. Propranolol inhibited stress-induced tumor growth and prolonged survival of the chronically stressed mice possibly by counteracting the above-mentioned effects [75]. Similarly, Li and Xu showed that propranolol blocked the effect of fear stress on tumor growth in PC xenograft animal models by decreasing Frizzled-1 (Fz1), Wnt-1 and vimentin protein expression [76]. In addition, Bernhard W. Renz and colleagues showed that sympathectomy (ganglionectomy) of the pancreas of KPC pancreatic cancer animal models, combined with gemcitabine, significantly improved mice survival, supporting the notion that locally delivered catecholamines contribute to cancer growth [72]. Consistently, propranolol decreased viability in three primary human PC organoids, especially when combined with gemcitabine, supporting the relevance of β-adrenergic signaling and nerve–cancer interactions in PC [72]. Moreover, the drug prolonged the survival of KPC mice by reducing tumor growth, invasiveness and cancer-related immunosuppression through β2-adrenergic receptor (ADRB2) blockade. In fact, ADRB2 is significantly upregulated in these genetically engineered pancreatic cancer mouse models, thereby increasing NGF and BDNF production, stimulating NGF/Trk pathways and enhancing pancreatic nerve density [72]. A further study by Al-Wadei et al. showed that propranolol prevents PC development in hamsters with ethanol-induced pancreatitis by nicotine-derived nitrosamine (NNK), a beta-adrenergic agonist, through a mechanism involving the block of cAMP-dependent release of EGF and VEGF, the down-regulation of the α7nicotinic acetylcholine receptor (α7nAChR), as well as the extracellular signal regulated protein kinases (ERK1/2) and p-CREB [77]. The impact of BBs was further retrospectively analyzed in a cohort of patients with stage II/III pancreatic adenocarcinoma. These patients, undergoing surgery and receiving non-selective BBs, including propranolol, showed fewer intratumoral S-100+ structures (a reduced nerve density), decreased staining for BDNF, a trend toward a lower perineural invasion/nerve and better survival, as compared with patients not receiving BBs, or receiving selective B1Bs, thus supporting a correlation between ADRB2 blockade and clinical outcome in PC [72].

The PROSPER trial is an ongoing phase II randomized study that will assess a combination of propranolol and etodolac used perioperatively for a total of 25 days in patients with resectable PC. The primary endpoint of the study is safety and feasibility of this approach. As a secondary objective, the trial will assess the utility of the two drugs in improving survival. The study includes an ancillary translational section to assess the mechanism of action of the combination [91].

## 3. Molecular Targets for Repurposed Drug Candidates: Hints from an In Silico Analysis

As summarized in Table 1, repurposed drugs have the great potential to impact multiple molecular targets implicated in PC biology, both in cancer cells and in components of tumor microenvironment. They are able to modulate metabolic reprogramming, immune system changes and cancer stemness pathway activation responsible for acquired resistance to standard chemotherapy. Indeed, it is conceivable that other targets, in addition to those already validated, may play an important role in the antineoplastic effects of repurposed drug candidates. An exploratory analysis that we conducted using the publicly available web tool SwissTargetPrediction, to gain further insights into the potential of the bioactive small molecules discussed in the review, suggests that there are additional, unexplored, oncology-relevant targets that may play a significant anticancer role. This bioinformatic approach, also known as target fishing, allows us to assess important information on the chemical structure of these agents, according to computed descriptors [221]. Moreover, the tool enables us to assess a broader polypharmacological profile of the repurposed drugs and to forecast side effects. Specifically, our analysis unraveled known and alternative target(s) for repurposed drugs outlined in this review, based on highly performant in silico predictions and similarity principles. In Table 3, for each repurposed drug, we report the main predicted human targets having probability scores higher than 0.6. The probability score is directly provided and calculated by the software. A value of 1 corresponds to 100% probability, whereas a value >0.6 corresponds to a probability score higher than 60%. The threshold indicated was chosen to limit the number of targets calculated by the software. Thus, we omitted targets with a score below 0.6 in order to provide more reliable information for suggesting useful pharmacological evaluations. Notably, for most compounds, we obtained results endowed with high probability scores. It is worth noting that predicted targets may or may not be implicated in cancer biology and, intriguingly, some of them could be also considered as potential off-targets relevant for cancer therapy. Future studies will be necessary to validate the possible interactions suggested by the tool between the repurposed drugs and the predicted novel targets in PC.

## 4. Immune-Modulating Potential of Repurposing Drug Candidates

A scorching field of study that is emerging regards the immune-modulating potential of repurposing drug candidates. Therapeutic strategies based on activation of antitumor immune response, such as immune checkpoint inhibition, achieve remarkable long-term remissions in some responsive tumors, albeit only a fraction of patients with advanced tumors respond to these therapies. PC is particularly unresponsive to these therapeutic approaches and drugs able to sensitize tumor cells to immunomodulating therapies are highly needed in PC patients and in other patients refractory to these therapeutic strategies. As reported in this review, some repurposing drug candidates in PC, such as chloroquine, metformin, losartan and propranolol appear to have beneficial effects on antitumor immune response. Each of these drugs acts with distinct mechanisms. Inhibition of autophagy by chloroquine, or by genetic approaches, was shown to be effective in restoring major histocompatibility complex (MHC) class I expression at the PC cell surface, thus reverting one of the main mechanisms of PC immune escape [50]. In fact, MHC-I molecules were shown to be mostly sequestered in autophagosomes in PC cells, which in turn hampers tumor antigen recognition by T-cells and contributes to immune escape of PC cells [50]. Autophagy inhibition by chloroquine enhanced the antitumor response to dual immune checkpoint blockade (ICB) therapy, including anti-CTL4 and anti-PD-1, in a mouse model of PC, although the combined treatment was able to slow down tumor growth, but it did not lead to complete tumor eradication [50]. Moreover, tumors treated with chloroquine and ICB had increased cytotoxic T-cell infiltration, supporting the notion that autophagy is a key regulator of immunogenicity in PC cells. In analogy to chloroquine, metformin also enhances response to immune checkpoint blockade (ICB) therapy in preclinical models [172]. In particular, metformin-activated AMPK appears to be effective in suppressing PD-L1/PD-1 signaling pathways via endoplasmic-reticulum-associated degradation of PD-L1 in different in vitro and in vivo tumor models [172]. In melanoma, breast and colon cancer, metformin had synergistic effects in combination with an anti-CTLA4 treatment [172]. The immunomodulatory effects of metformin in PC are not known and might represent an additional field of study of this poorly immunogenic tumor. AT1R blockade by losartan was shown to decrease TANs and regulatory T cells (Tregs) in PC TME, along with the increase in CD8+ T cells, through the prevention of PSC activation and subsequent reduction in IL-1β expression [58]. Similarly, losartan was able to inhibit aberrant TGF-β activity, followed by reduced collagen deposition and accumulation of Tregs in an orthotopic model of pancreatic cancer [59]. Taken together, these findings suggest a potential role of losartan in reversing the typical immunosuppressive PC microenvironment, which warrants further investigation. Propranolol reduces the infiltration of myeloid cells, particularly neutrophils, and increases cytotoxic tumor infiltrating lymphocytes in the tumor stroma of melanoma mouse models, which are beneficial in restoring a better control of the tumor by cytotoxic cells [222]. These effects on tumor infiltrating immune cells deserve further investigations in PC.

Among additional repurposing drug candidates that could improve anti-tumor immune response, it was reported that, in analogy to PCSK9 deletion, PCSK9-neutralizing antibodies, clinically approved for hypercholesterolemia treatment, synergize with anti-PD1 therapy in different mouse cancer models. PCSK9-neutralizing antibodies increase the expression of MHC-I proteins by promoting their recycling to the surface of tumor cells and, therefore, opposing their lysosomal degradation [223]. Considering that PCSK9 levels are inversely correlated with markers of intratumoral T-cell infiltration and survival in PC patients, it will be interesting to study whether the combination of PCSK9-neutralizing antibodies with immune checkpoint blockade may be beneficial also in PC.

## 5. Concluding Remarks and Future Perspectives

Repurposing non-oncology drugs in cancer therapy represents a promising and valuable therapeutic opportunity, especially in patients with advanced disease, or chemo-resistant tumors that lack alternative therapeutic options, as in the case of pancreatic cancer. In the present review, we outlined the antitumor properties displayed by different non-oncology drugs in preclinical and clinical studies carried out in PC. As discussed, repurposed drugs have the great potential to modulate multiple pathways and molecular targets known or previously unknown to be crucial in cancer biology, possibly with improved effectiveness and ability to overwhelm resistance to standard chemotherapy drugs. Moreover, it is likely that drug combinations including repurposed drug candidates may act as new entities exerting antineoplastic activities distinct from those shown by individual drugs, through the modulation of additional cancer-relevant pathways. This possibility may represent an important field in the search for new, more effective strategies in PC treatment. As mentioned above, another exciting field of study concerns the immune-modulating potential of repurposing drug candidates. PC is particularly unresponsive to immunotherapeutic approaches, one of the most effective therapeutic strategies in other advanced tumors. Some repurposing drug candidates, such as chloroquine, metformin, losartan and propranolol, appear to have beneficial effects on antitumor immune response through distinct mechanisms. Some of these drugs were shown to enhance response to therapy with immune checkpoint inhibitors in preclinical models. This is particularly relevant, as it may expand their potential clinical application in synergy with approved anticancer immunomodulatory agents that are mostly ineffective as single agents in PC.

One of the limitations of non-oncology drugs candidates for repurposing as anticancer treatments is related to the dosages used in preclinical studies to obtain antitumor effects, which may exceed the doses required for the original indication. However, the need for higher dosages does not necessarily mean that the higher dosage is toxic in humans, but a dose-finding trial is required in the target population for safety assessment. Moreover, combination therapies with other agents in principle may lower the doses required to attain antitumor effects and this may circumvent the problem of increasing the original dosages of each drug [224]. Additional issues that repurposed drug candidates face in view of their successful translation in clinical setting include, but are not limited to, legal and safety liabilities, intellectual property rights, patent and regulatory barriers, together with the relative lack of funding for clinical trial institution by pharmaceutical companies because of expected low returns on investment [11,19,225]. In this regard, repurposing trials are often managed by non-pharmaceutical industries, such as non-profit organizations, hospitals or academic groups, which may have limited knowledge of stringent regulatory needs for label extension, and this may complicate implementation of promising repurposed drug candidates in clinical settings [11,19].

In conclusion, drug repurposing represents an attractive option that is worth exploring in oncology, especially in the case of tumors lacking successful therapeutic strategies, such as pancreatic cancer. As outlined in this review, there are several non-oncology drug candidates for repurposing in pancreatic cancer, some of which are currently being tested in clinical trials. However, further clinical studies will be necessary in view of their potential clinical adoption.

## Figures and Tables

**Table 1:** Repurposing drug candidates and proposed molecular pathways they modulate in pancreatic cancer.

Drug Name	Original Indications	Type of Study	Main Results of the Study (Proposed Molecular Targets/Mechanisms of Action in PC)	Refs.
Auranofin	Rheumatoid arthritis	*In vitro/* *animal models*	Inhibition of TrxR1 and HIF1α leading to decreased antioxidant activity within PC cells, followed by apoptosis; antitumor effect at the primary site and suppression of distant organ metastasis in PC orthotopic mouse models	[20]
		*In vitro/* *animal models*	Preferential cytotoxicity towards PC cells under nutrient-deprived conditions through ROS accumulation and subsequent induction of apoptosis by caspase 3/7 activation and proteolytic PARP cleavage; suppression of tumor growth in a PC xenograft model	[21]
		*In vitro/* *animal models*	Increase in mitochondrial ROS and apoptosis, together with inhibition of autophagic flux when used in combination with an engineered human cyst(e)inase; suppression of tumor growth in PC xenografts by combined treatment	[22]
Haloperidol	Psychosis	*In vitro/* *animal models*	Blockade of DRD2 leading to inhibition of proliferation by promoting ER stress, impairment of migration and cell cycle progression, induction of apoptosis in PC cells; reduction in tumor size and metastatic dissemination in mice with orthotopic xenograft PC tumors	[23]
		*In vitro*	Inhibition of PC cell viability by restoring the expression of *DUSP6* gene through epigenetic modification of its transcriptional regulation	[24]
Penfluridol	Psychosis	*In vitro/* *animal models*	Inhibition of PC cell proliferation by inducing ER stress, leading to autophagy and apoptosis; reduction in tumor growth in xenograft and orthotopic PC models	[25,26]
		*In vitro*	Inhibition of proliferation, promotion of cell cycle arrest and apoptosis in PC cells through the activation of PP2A; synergistic effects on the viability of gemcitabine-resistant and gemcitabine-sensitive PC cells when used in combination with gemcitabine	[27]
		*In vitro/* *animal models*	Inhibition of JAK2–STAT3 and ERK/AKT signaling, through binding with the JAK2-binding site in PRLR, leading to suppression of colony and spheroid formation, together with induction of autophagy; slowdown of tumor growth in different xenograft mouse models of PC	[28]
Parbendazole	Intestinal parasitic infections	*In vitro*	Inhibition of proliferation, clonogenicity and migration of PC cells, together with promotion of apoptosis, cell cycle perturbation and DNA damage response through the alteration of microtubule organization, formation of irregular mitotic spindles and appearance of polyploid cells; synergistic effects on PC cell viability when used in combination with gemcitabine	[29]
Disulfiram	Chronic alcoholism	*In vitro/* *animal models*	A complex formed by a disulfiram metabolite bound to copper (DDTC-Cu) induces inhibition of PC cell proliferation through the impairment of proteasome activity; reduction in tumor growth in xenograft mouse models of PC, with accumulation of ubiquitinated proteins, up-regulation of p27 and down-regulation of NF-kB expression in tumor tissues	[30]
		*In vitro/* *animal models*	A complex formed by a disulfiram metabolite bound to copper (DDTC-Cu) induces depletion of pre-existing CSCs and radiation-induced CSCs in PC cells through NF-kB-stemness gene pathway downregulation; antitumor effects in syngeneic mouse PC models, with reduced sphere formation, when used in combination with 5-FU and radiotherapy	[31]
		*In vitro/* *animal models*	Promotion of aponecrosis death pathways in K-Ras mutant PC cell lines, through intracellular ATP depletion and ROS release, when used in combination with arsenic trioxide and ascorbic acid; reduction in tumor growth in mice with PANC-1 xenografts by using the three-drug combination	[32]
		*In vitro*	Induction of autophagy-dependent apoptosis in PC cells through the activation of the ER stress/IRE1α-XBP1 pathway, by a direct interaction with IRE1α, or by an indirect mechanism involving the inhibition of the NPL4 cofactor of the p97/VCP segregase and of proteasome, along with the production of ROS	[33]
Doxycycline	Bacterial infections	*In vitro/* *animal models*	Inhibition of PC cell proliferation by activating proapoptotic genes and by suppressing antiapoptotic genes, perturbating cell cycle and inhibiting the expression of the proangiogenic IL-8; reduction in tumor growth by 80% in a xenograft mouse model of PC	[34,35]
		*In vitro*	Inhibition of tumorsphere formation in PC cells, without affecting the viability of both bulk cancer cells and normal fibroblasts	[36]
		*In vitro*	Inhibition of mitochondrial protein synthesis leading to decreased PC cell proliferation, through ATP depletion, when used in combination with gemcitabine; enhancement of gemcitabine-induced apoptosis by decreasing mitochondrial membrane potential and fostering ROS production	[37]
		*In vitro/* *animal models*	Inhibition of PC cell growth, migration, invasion and tumorsphere formation through the down-regulation of PAR1/FAK/PI3K/AKT signaling pathway; synergistic effects on PC cell viability and reduction in tumor growth by 80.5% in subcutaneous Panc-1 xenografts models, when used in combination with 5-FU, with the increase in E-Cadherin expression and the decrease in Vimentin and CD133 expression	[38]
Efavirenz	HIV infection	*In vitro*	Impairment of clonogenicity and induction of apoptosis in distinct PC cell lines	[39]
		*In vitro*	Inhibition of PC cell proliferation through a mechanism involving ROS production and mitochondrial membrane depolarization, along with phosphorylation of both ERK1/2 and p38 MAPK stress pathways, when used in combination with radiation	[40]
Nelfinavir	HIV infection	*In vitro/* *animal models*	Inhibition of Akt phosphorylation in PC cells leading to enhanced radiosensitization of both wild-type and K-ras mutant cell lines; synergistic slowdown of tumor growth in Capan-2-bearing xenografts, when used in combination with radiation	[41]
		*In vitro*	Impairment of clonogenicity and viability in distinct PC cells by affecting cell cycle and promoting apoptosis; synergistic antitumor effects in PC cells when used in combination with nitroxoline	[42]
Ritonavir	HIV infection	*In vitro*	Inhibition of PC cell viability by triggering the intrinsic apoptotic pathway and interfering with cell cycle machinery through the suppression of Akt and Rb phosphorylation in cells; impairment of PC cell motility and invasiveness; enhancement of antiproliferative effects in PC cells, when used in combination with gemcitabine	[43]
(Hydroxy)-Chloroquine	Malaria Systemic Lupus Erythematosus Rheumatoid arthritis	*In vitro/* *animal models*	Inhibition of proliferation in several PC cell lines; improvement of survival in murine xenograft models of PC	[44]
		*In vitro/* *animal models*	Inhibition of autophagy in PSCs through reduced IL-6 expression and ECM protein production, leading to a quiescent state of PSCs; attenuation of invasion properties and liver metastasis formation in an orthotopic PC mouse model	[45]
		*In vitro*	Inhibition of viability in metastatic PC cell lines, with enhanced effects in hypoxia	[46]
		*In vitro*	Enhancement of antiproliferative effects of 5-fluorouracil, or gemcitabine by reversing autophagy-mediated cytoprotective mechanisms induced by chemotherapy drugs	[47]
		*In vitro/* *animal models*	Inhibition of autophagy leading to increased gemcitabine-induced cytotoxicity through ROS release, lysosomal membrane permeabilization and subsequent apoptosis in PC cells; reduction in tumor growth in xenograft PC models, when used in combination with gemcitabine	[48]
		*In vitro/* *animal models*	Enhancement of antitumor effects in both organoid and PDX models of PC, when used in combination with ERK/MAPK inhibitors, which induce a state of autophagy-dependence in PC cells due to impaired mitochondrial functions and glycolytic activity	[49]
		*In vitro/* *animal models*	Inhibition of autophagy leading to restoring MHC class I expression at the PC cell surface and reverting one of the PC immune escape mechanisms; slowdown of tumor growth in mouse model of PC, when used in combination with dual immune checkpoint blockade inhibitors (anti-CTL4 and anti-PD-1)	[50]
		*In vitro/* *animal models*	Inhibition of CXCL12/CXCR4 signaling, reduced phosphorylation of ERK and STAT3, downregulation of Hedgehog signaling, leading to elimination of Pa-CSCs; reduction in tumorigenicity and invasiveness in pancreatic cancer PDX models; improved outcomes of mice bearing primary xenografts, when used in combination with gemcitabine	[51]
Itraconazole	Fungal infections	*In vitro/* *animal models*	Inhibition of PC cell proliferation by induction of apoptosis through ROS release, mitochondrial membrane depolarization and Bak-1 activation; inhibition of PC xenograft tumor growth	[52]
		*In vitro/* *animal models*	Inhibition of PC cell viability by apoptosis induction, together with impairment of migration, invasion and EMT of PC cells through TGF-β/SMAD2/3 signaling suppression; inhibition of tumor growth in GEMM of PC	[53]
Losartan	Hypertension	*In vitro/* *animal models*	Blockade of AT1R leading to inhibition of VEGF synthesis and suppression of PC cell proliferation; improved survival benefit in rat orthotopic PC models, when used in combination with gemcitabine	[54]
		*In vitro/* *animal models*	Inhibition of PC growth in vivo by sequential administration of losartan- followed by gemcitabine-loaded magnetic mesoporous organosilica spheres leading to reduced stromal type I collagen and hyaluronic acid components in the ECM of the tumor	[55]
		*In vitro/* *animal models*	Blockade of AT1R leading to impaired stromal collagen and hyaluronan production by CAFs; improved overall survival in orthotopic PC mouse models through enhanced tumor perfusion, oxygen, chemo- and nanotherapeutics delivery	[56,57]
		*Animal models/* *in patients*	Blockade of AT1R leading to attenuated obesity-induced fibrosis, tumor progression and improved response to chemotherapy by preventing PSC activation, increasing CD8+ T cells, decreasing IL-1β, TANs and Tregs	[58]
		*Animal models*	Slowdown of PC progression and improved survival in orthotopic models of PC by inhibiting aberrant TGF-β activity, collagen deposition and accumulation of Tregs	[59]
Metformin	Type 2 diabetes	*In vitro*	Inhibition of respiratory complex I leading to reduced ATP production, increased AMP/ATP ratio, activation of AMPK signaling, followed by downregulation of mTOR, which inhibits PC cell proliferation	[60]
		*In vitro/* *animal models*	Inhibition of PC cell proliferation through the suppression of insulin/IGF-I receptor activation and downstream signaling mediators IRS-1 and Akt; inhibition of tumor growth in PC xenografts mouse models	[61,62]
Nitroxoline	Urinary tract infections	*In vitro*	Inhibition of viability and clonogenicity, induction of cell cycle arrest and apoptosis, impairment of migration and invasion in PC cells through deregulation of Na/KATPase pump, β-catenin pathway, cytosolic iron homeostasis, together with ROS production and mitochondrial depolarization; synergistic antitumor effects in PC cells when used in combination with nelfinavir	[42,63]
Pimavanserin	Parkinson disease psychosis	*In vitro/* *animal models*	Inhibition of viability, promotion of apoptosis and suppression of tumorsphere formation in PC cells through the abrogation of Akt/Gli1 signaling cascade leading to the downregulation of Oct-4, SOX2 and NANOG cancer stem cell markers; reduction in tumor growth in both subcutaneous and orthotopic xenografts models of PC	[64]
Pirfenidone	Idiopathic pulmonary fibrosis	*In vitro*	Inhibition of proliferation and promotion of cell cycle arrest in both PSCs and PC cells	[65]
		*In vitro/* *animal models*	Inhibition of proliferation, invasion and migration of PSCs through the suppression of PDGF-A, HGF, periostin, collagen type I and fibronectin; suppression of PC growth and metastasis in mice co-implanted with PC cells and PSCs by disrupting tumor-stromal interactions	[66]
		*Animal models*	Inhibition of desmoplastic reactions and tumor growth in HapT1-derived orthotopic hamster PC models by reducing αSMA-positive cells and collagen deposition	[67]
		*In vitro*	Inhibition of CHI3L1 and FN1, leading to reversion of gemcitabine resistance in PC cells	[68]
		*In vitro/* *animal models*	A liposome-based nanomedicine integrating pirfenidone and gemcitabine induces downregulation of collagen I and TGF-β in PSCs; slowdown of tumor growth in mice co-implanted with PC cells and PSCs through decreased stromal fibrosis and increased drug perfusion	[69,70]
		*In vitro/* *animal models*	Organosilica nanoparticles integrating pirfenidone and gemcitabine induce inhibition of PC cell viability by apoptosis induction, together with reduction in collagen I and fibronectin ECM components; reduction in tumor growth in mice co-implanted with PC cells and PSCs through decreased collagen I and fibronectin and enhanced endovascular osmotic pressure	[71]
Propranolol	Hypertension	*In vitro/* *animal models/* *in patients*	Inhibition of viability in primary human PC organoids, as a single agent and in combination with gemcitabine; abrogation of tumor growth, invasiveness and cancer-related immunosuppression, together with prolonged survival in KPC mice through ADRB2 blockade; improved clinical outcome in patients with stage II/III pancreatic adenocarcinoma undergoing surgery and receiving non-selective BBs, showing reduced nerve density, lower perineural invasion and decreased staining for BDNF (retrospective data)	[72]
		*In vitro/* *animal models*	Blockade of B1/B2-adrenoceptors leading to suppression of PC cell proliferation and invasiveness by inducing apoptosis and by inhibiting the expression of NF-kB, AP-1 and CREB, as well as the expression of MMP-9, MMP-2 and VEGF target genes	[73,74]
		*Animal models*	Blockade of B1/B2-adrenoceptors leading to inhibition of tumor growth and prolonged survival in chronically stressed immunocompetent orthotopic syngeneic murine model of PC	[75]
		*Animal models*	Blockade of B1/B2-adrenoceptors leading to inhibition of stress-induced tumor growth in PC xenograft animal models by decreasing Fz1, Wnt-1 and vimentin expression	[76]
		*Animal models*	Prevention of PC development in hamsters with ethanol-induced pancreatitis by NNK, through the block of cAMP-dependent release of EGF and VEGF, together with the down-regulation of α7nAChR, ERK1/2 and p-CREB	[77]

Abbreviations: Thioredoxin reductase 1, TrxR1; Hypoxia-inducible factor-1 α, HIF1α; Reactive oxygen species, ROS; Poly (ADP-ribose) polymerase, PARP; Dopamine D2 receptor, DRD2; Endoplasmic reticulum, ER; Dual-specificity phosphatase 6, DUSP6; Protein phosphatase 2A, PP2A; Janus kinase 2-signal transducer and activator of transcription 3, JAK2–STAT3; Extracellular signal-regulated kinase/Protein kinase B, ERK/AKT; Prolactin receptor, PRLR; Diethyldithiocarbamate/copper, DDTC-Cu; Nuclear factor kB, NF-kB; Cancer stem cells, CSCs; 5-fluorouracil, 5-FU; Endoribonuclease inositol-requiring enzyme 1 α, IRE1α; X-Box Binding Protein 1, XBP1; Nuclear protein localization protein 4, NPL4; Human ubiquitin-selective protein segregase p97 (also known as VCP; valosin-containing protein), p97/VCP segregase; Human immunodeficiency virus, HIV; Mitogen-activated protein kinases, MAPK; Retinoblastoma protein, Rb; Pancreatic stellate cells, PSCs; Extracellular matrix, ECM; Patient-derived xenografted, PDX; Major histocompatibility complex, MHC; Cytotoxic T-Lymphocyte Antigen 4, CTL4; C-X-C Motif Chemokine Ligand 12/C-X-C chemokine receptor type 4, CXCL12/CXCR4; Pancreatic cancer stem cells, Pa-CSCs; BCL2 Antagonist/Killer 1, Bak-1; Epithelial to mesenchymal transition, EMT; Transforming growth factor beta/small mother against decapentaplegic2/3, TGF-β/SMAD2/3; Genetically engineered mouse model, GEMM; Angiotensin II type 1 receptor, AT1R; Vascular endothelial growth factor, VEGF; Cancer-associated fibroblasts, CAFs; Interleukin 1 beta, IL-1β; Tumor-associated neutrophils, TANs; Regulatory T cells, Tregs; Transforming growth factor-β, TGF-β; AMP kinase, AMPK; Mammalian target of rapamycin, mTOR; Insulin-like growth factor-I, IGF-I; Insulin receptor substrate 1, IRS-1; Protein kinase B/glioma-associated oncogene homolog 1, Akt/Gli1; octamer-binding transcription factor 4, Oct-4; SRY-Box Transcription Factor 2, SOX2; Homeobox protein, NANOG; Platelet-derived growth factor subunit A, PDGF-A; Hepatocyte growth factor, HGF; α smooth muscle actin, αSMA; Chitinase 3-like-1, CHI3L1; Fibronectin, FN1; Activator protein 1, AP-1; cAMP response element binding protein, CREB; Matrix metalloproteinase 9, MMP-9; Matrix metalloproteinase 2, MMP-2; Frizzled-1, Fz1; Genetically Engineered Mouse Models of Pancreatic Cancer, KPC mice; B2-adrenergic receptor, ADRB2; Beta-blockers, BBs; Brain-derived neurotrophic factor, BDNF; Nicotine-derived nitrosamine, NNK; Epidermal growth factor, EGF; α7nicotinic acetylcholine receptor, α7nAChR.

**Table 2:** Overview of interventional clinical trials investigating repurposed drugs in PC.

Repurposed Drug	Register Trial Code/ Study Reference	Phase	Interventions	*n*. Patients	Status/Results
Chloroquine/ Hydroxychloroquine	NCT01777477 [78]	I	GEM + Chloroquine in mPC	9	Safe; 1 PR; 2 SD mTTP: 4 mo mOS: 7.6 mo
	NCT01128296 [79]	I/II	Neoadjuvant GEM + Hydroxychloroquine in rPC	35	Safe; 94% resection rate 77% RO resection rate OS and DFS correlated with LC3-II expression in circulating PBMC
	NCT01978184 [80]	Randomized II	Neoadjuvant GEM/nabP ± hydroxychloroquine in rPC/brPC	64	Evans II grade response rate: 55.9% vs. 10%
	NCT01506973 [81]	Randomized II	GEM/nabP ± hydroxychloroquine in untreated laPC/mPC	112	12-month OS: 41% vs. 49% ORR:38.2% vs. 21.1% mOS: 11.1 vs. 12.1 mo
	NCT04132505	I	Binimetinib + hydroxychloroquine in mPC	39	Recruiting
	NCT03825289	I	Trametinib + Hydroxychloroquine in aPC/mPC	33	Recruiting
	NCT04386057	Randomized II	LY3214996 ± hydroxychloroquine in pretreated laPC/mPC	52	Recruiting
Disulfiram	NCT02671890	Partially randomized I	Disulfiram in refractory/ GEM-pretreated mPC	74	Recruiting
	NCT03714555	II	Disulfiram + copper gluconate in mPC treated with FOLFIRINOX, GEM/nabP or GEM and with rising CA19.9	42	Recruiting
Nelfinavir	[82]	I	Nelfinavir + Chemoradiation in locally laPC	12	Safe
	NCT01068327 [83]	I	Nelfinavir + SBRT in brPC/laPC	46	Safe
	[84]	II	Nelfinavir + chemoradiation in laPC	23 (prematurely closed due to drug unavailability)	1yr-OS: 73.4% mOS:17.4 mo 1-yr PFS:21.8% mPFS:5.5 mo
	[85]	I/II	Oregovomab + SBRT + Nelfinavir in laPC	11 (prematurely closed due to changed standard of treatment)	Safe
	NCT02024009 [86]	Randomized II	GEM/nab followed by chemoradiation ± nelfinavir in laPC	289	Recruiting
Losartan	NCT01821729 [87]	II	Losartan + FOLFIRINOX + chemoradiation in laPC	49	R0 resection rate: 65%
	NCT04539808	II	Neoadjuvant FOLFIRINOX/GEM-nabP + chemoradiation + losartan in rPC/brPC	40	Recruiting
Metformin	NCT01971034 [88]	II	Metformin + paclitaxel in laPC/mPC refractory to GEM	20	Terminated for futility
	NCT01210911 [89]	Randomized II	GEM/erlotinib ± metformin in laPC/mPC	121	mOS: 56.7% vs. 63.9% (n.s.)
	NCT01167738 [90]	Randomized II	Cisplatin/epirubicin/capecitabine/GEM ± metformin in mPC	60	Terminated for futility
	NCT02336087	I	GEM/nabP + dietary supplements + metformin in laPC	21	Active, not recruiting
	NCT01666730	II	FOLFOX6 + metformin in mPC	50	Completed, results not published
	NCT02153450	I	SBRT + metformin in rPC/laPC	8	Completed, results not published
	NCT02005419	II	Metformin + GEM in resected PC	300	Completed, results not published
Propranolol	EudraCT 2018-000415-25 [91]	Randomized II	Perioperative propranolol + etodolac vs. placebo in rPC	80	Recruiting

**Abbreviations:** rPC = resectable Pancreatic cancer; brPC = borderline resectable Pancreatic Cancer; laPC = locally advanced Pancreatic Cancer; mPC = metastatic Pancreatic Cancer; PR = Partial Response; SD = Stable Disease; mPFS = median Progression Free Survival; mOS = median Overall Survival; mTTP: median Time to Progression; ORR = Overall Response Rate; GEM = Gemcitabine; nabP = nab-Paclitaxel; SBRT = Stereotactic Body Radiotherapy.

**Table 3:** Predicted molecular targets and probability scores (≥0.6) for each repurposed drug according to the SwissTargetPrediction tool.

Repurposed Drug	Predicted Targets (Probability Score)
Auranofin	—
Haloperidol	5-HT2b receptor (1), Dopamine D5 receptor (1), Alpha-2a adrenergic receptor (1), Adrenergic receptor alpha-2 (1), Histamine H2 receptor (1), Alpha-2b adrenergic receptor (1), Muscarinic acetylcholine receptor M5 (1), Dopamine D1 receptor (1), Muscarinic acetylcholine receptor M2 (1), Serotonin 1a (5-HT1a) receptor (1), Muscarinic acetylcholine receptor M1 (1), Dopamine D2 receptor (1), Dopamine D4 receptor (1), Norepinephrine transporter (1), Alpha-1d adrenergic receptor (1), Serotonin 2a (5-HT2a) receptor (1), Serotonin 2c (5-HT2c) receptor (1), Serotonin transporter (1), Alpha-1a adrenergic receptor (1), Histamine H1 receptor (1), Potassium channel subfamily K member 2 (1), Mu opioid receptor (1), Dopamine D3 receptor (1), HERG (1), Sigma opioid receptor (1), Serotonin 7 (5-HT7) receptor (1), Serotonin 6 (5-HT6) receptor (1), Histone H1.0 (1), P-glycoprotein 1 (1), Anti-estrogen binding site (AEBS) (1)
Penfluridol	—
Parbendazole	—
Disulfiram	Alpha-2a adrenergic receptor (1), Alpha-2b adrenergic receptor (1), Dopamine D1 receptor (1), Dopamine D2 receptor (1), Dopamine D4 receptor (1), Mu opioid receptor (1), Dopamine D3 receptor (1), Kappa Opioid receptor (1), Dopamine transporter (1), C-C chemokine receptor type 4 (1), Interleukin-8 receptor B (1), Adenosine A3 receptor (1), Cytochrome P450 1A2 (1), Serotonin 6 (5-HT6) receptor (1), MAP kinase ERK1 (1), C-C chemokine receptor type 2 (1), Monoglyceride lipase (1), Serotonin 1b (5-HT1b) receptor (1)
Doxycycline	—
Efavirenz	—
Nelfinavir	Neurokinin 2 receptor (0.97), Mu opioid receptor (0.97), Tyrosine-protein kinase FYN (0.74), Dopamine D1 receptor (0.74), Norepinephrine transporter (0.74), Adenosine A3 receptor (0.74), Cytochrome P450 3A4 (0.74)
Ritonavir	Cytochrome P450 3A4 (0.64), Neurokinin 2 receptor (0.62)
Chloroquine	HERG (1), Quinone reductase 2 (1), Prion protein (1), Histamine N-methyltransferase (0.67), Histamine H3 receptor (0.67)
Hydroxychloroquine	Muscarinic acetylcholine receptor M2 (1), Alpha-1d adrenergic receptor (1)
Itraconazole	—
Losartan	Type-1 angiotensin II receptor (1), Angiotensin converting enzyme (0.91), Cytochrome P450 2C9 (0.91)
Metformin	—
Nitroxoline	Cyclooxygenase-2 (1), Methionine aminopeptidase 2 (1)
Pimavanserin	—
Pirfenidone	—
Propranolol	Serotonin 2b (5-HT2b) receptor (1), Serotonin 1b (5-HT1b) receptor (1), Adrenergic receptor beta (1), Beta-1 adrenergic receptor (1), Serotonin 1a (5-HT1a) receptor (1), Serotonin 2a (5-HT2a) receptor (1), Serotonin 2c (5-HT2c) receptor (1), Serotonin transporter (1), Beta-3 adrenergic receptor (1), Sigma opioid receptor (1), Cytochrome P450 2D6 (1), Cytochrome P450 1A2 (1), Serotonin 6 (5-HT6) receptor (1)

(—) indicates that molecular targets predicted by SwissTargetPrediction tool have probability scores <0.6.
